# Chaos Detection by Fast Dynamic Indicators in Reflecting Billiards

**DOI:** 10.3390/e25091251

**Published:** 2023-08-23

**Authors:** Gabriele Gradoni, Giorgio Turchetti, Federico Panichi

**Affiliations:** 1 Department of Electrical and Electronic Engineering, School of Mathematical Sciences, University of Nottingham, University Park, Nottingham NG7 2RD, UK; 2 Department of Physics and Astronomy, Alma Mater Studiorum, University of Bologna, Viale Berti Pichat 6/2, 40127 Bologna, Italy; 3 INdAM Gruppo Nazionale per la Fisica Matematica Piazzale Aldo Moro, 00185 Roma, Italy

**Keywords:** Lyapunov error, reversibility error, Gibbs entropy, wave chaos

## Abstract

The propagation of electromagnetic waves in a closed domain with a reflecting boundary amounts, in the eikonal approximation, to the propagation of rays in a billiard. If the inner medium is uniform, then the symplectic reflection map provides the polygonal rays’ paths. The linear response theory is used to analyze the stability of any trajectory. The Lyapunov and reversibility error invariant indicators provide an estimate of the sensitivity to a small initial random deviation and to a small random deviation at any reflection, respectively. A family of chaotic billiards is considered to test the chaos detection effectiveness of the above indicators.

## 1. Introduction

Electromagnetic cavities exhibit wave chaos that can be predicted by a semi-classical analysis and random matrix theory; see [1] for a two-dimensional open electromagnetic cavity, ref. [2] for closed three-dimensional cavities and [3] for a review. These powerful prediction tools hold true and can be extended to include coupling through antennas and wave-guides, as well as interconnected cavities [4]. Furthermore, the semi-classical treatment of electromagnetic cavities has been tackled extensively in [5,6]. The experimental verification of wave chaos in microwave billiards has been addressed by several international research groups over the last few decades. This extensive work led to the observation of a rich phenomenology originating from complex wave dynamics [7], including: Wave-function scars [8], chaotic dynamics in superconducting billiards [9], time-reversal symmetry breaking [10], nodal domains in rough billiards [11], time-invariance violation at and around exceptional points [12] as well as electromagnetic reverberation [13]. Concerning optical resonators, it has been shown that chaotic dynamics are a key mechanism in asymmetric geometries [14,15,16]. This effect is important in the generation of lasers [17] with directional emissions [18] of eccentric cavities. Chaotic cavities have been employed in a plethora of electromagnetic engineering applications, including time reversal energy focusing [19] and electromagnetic compatibility (EMC) testing [20]. The equations for the propagation of acoustic and electromagnetic waves in resonant cavities are similar and coincide when the medium within the cavity is uniform. Usually, the cavities have a cylindrical or spherical symmetry and the resonant modes can be computed in a closed mathematical form. When the eikonal approximation is applicable, for sufficiently short wavelengths, the symplectic reflection map is integrable [21]. Deformations of the boundary cause the loss of integrability and the emergence of chaotic behavior in the particles or rays trajectories [22]. Even if the dynamics of integrable billiards are still an exciting research topic that continuously unveils novel phenomena, a growing interest has been developing within the scientific community for the properties of chaotic billiards [23,24,25,26,27].

For a sphere, the trajectory of any ray develops in a plane, which is determined by the initial ray position and velocity. The intersection of the sphere with the invariant plane is a circle, where the 2D area preserving the reflection map is integrable.

The symplectic 4D reflection map on a sphere is no longer integrable if the sphere is deformed. Thus, as opposed to a 4D flow, a first integral is no longer available and the 2D Poincaré map cannot be computed, preventing the analysis of the dynamical structures in phase plane portraits. For a cylinder, the trajectory of any ray develops in a plane, which intersects it on an ellipse, where the 2D reflection map is integrable. When the cylinder is deformed, the orbits no longer belong to a plane, the 4D symplectic map is not integrable and the phase portraits of the reflection map cannot be drawn [28]. This is important also in wave propagation within deformed cavities as the evolution of quantum states show footprints of classical trajectories [29].

The Lyapunov error ($E_{n}\left(x\right)$) and the reversibility error ($E_{n}^{R}\left(x\right)$) are dynamic indicators, based on the linear response theory, and allow the testing of the sensitivity of the orbits to small random deviations (see, for instance, [30]). For any fixed number of iterations, this sensitivity can be compared on a set of initial conditions chosen in a phase plane (see, for instance, [31]).

In Section 2, we present a brief introduction of the fast indicator used, their properties and relations. In Appendix B, we provide the definition of Lyapunov and reversibility errors. For a single orbit, the dependence on *n* can be investigated and the limit n→∞, limit of $n^{-1}\;\log E_{n}\left(x\right)$, provides the maximum Lyapunov exponent. $E_{n}^{R}\left(x\right)$ has been shown to be very sensitive to multi-dimensional problems, such as chaos detection in planetary systems (see, for instance, [32]) and in 2D and 3D waveguides (see [33]). In Section 3, we present a numerical analysis of the 2D reflection map in a convex domain, given by a deformed circle. We compare the phase portraits with the color plots of the Lyapunov and reversibility errors computed in a regular grid of phase space.

The mathematical description of the convex billiard and its new parametrization are presented in Appendix A.

To conclude, we consider the transport of particles and rays within the billiard. Given a source of particles or rays within the billiard, the time evolution of the probability density of particles or the energy density of rays is analyzed. Such a density can hardly be determined analytically, even for integrable billiards, and a numerical strategy is presented.

The dynamic indicators could be evaluated at time *t* for the inner points of the billiard after performing an average with respect to the initial ray or particle direction. A detailed analysis of 2D and 3D billiards can be worked out by using the algorithms described here.

## 2. Lyapunov and Reversibility Error Indicators

The dynamic stability of the reflection map can be analyzed with the Lyapunov error indicator (i.e., $E_{n}\left(x\right)$) or with the Reversibility error indicator (i.e., $E_{n}^{R}\left(x\right)$). The former measures the sensitivity of the initial conditions to small random deviations, and the latter measures the sensitivity to small deviations at each reflection. To implement the Reversibility error indicator, we iterate both the randomly perturbed and exact maps’ *n* times, and compute the distance of the final point of the perturbed orbit with respect to the one obtained from the exact orbit. Letting ϵ be the amplitude of the random perturbations, we consider the linear response given by the ϵ→0 limit. Given a symplectic or measure preserving map M(x), defined as a compact manifold of $R^{2d}$, we denote with DM(x) the tangent map where ${\left(DM\right)}_{ij}\left(x\right)=\partial \;M_{i}\left(x\right)/\partial x_{j}$. The orbit $x_{n}$ is obtained by iterating *n* times the map *M*. We introduce the matrix ${𝖫}_{n}$ obtained by taking the products of the tangent map along the orbit:

(1) $$\begin{array}{cc} \; & x_{n}=M\left(x_{n-1}\right)\;\;\;\;x_{0}=x\;\;x_{n}=M^{\circ n}\left(x\right)\\ \; & {𝖫}_{n}\left(x\right)=DM\left(x_{n-1}\right)\;{𝖫}_{n-1}\left(x\right)\;{𝖫}_{0}=𝖨\;\;{𝖫}_{n}\left(x\right)=DM^{\circ n}\left(x\right) \end{array}$$

For the initial condition y=x+ϵξ, where ***ξ*** is a random vector with a zero mean and unit covariance matrix $\langle \;\xi \;\xi^{T}\;\rangle =𝖨$, we compute the orbit $y_{n}=M\left(y_{n-1}\right)$ where $y_{0}=y$. We compare the perturbed and the reference orbit by defining the linear response ${𝜩}_{n}$ according to

(2) $$\begin{array}{cc} \; & {𝜩}_{n}\left(x\right)=\lim_{\epsilon \to 0}\;\frac{y_{n}-x_{n}}{\epsilon }=\lim_{\epsilon \to 0}\;\frac{M^{\circ n}\left(x+\epsilon \xi \right)-M^{\circ n}\left(x\right)}{\epsilon }=DM^{\circ n}\left(x\right)\;\xi \equiv {𝖫}_{n}\left(x\right)\;\xi \end{array}$$

The square of $E_{n}\left(x\right)$ is defined as the trace of the covariance matrix $\Sigma_{n}^{2}\left(x\right)$ of the random vector ${𝜩}_{n}\left(x\right)$:

(3) $$\begin{array}{c} \Sigma_{n}^{2}\left(x\right)=\langle \;{𝜩}_{n}\left(x\right)\;{𝜩}_{n}^{T}\left(x\right)\;\rangle ={𝖫}_{n}\left(x\right)\;{𝖫}_{n}^{T}\left(x\right)\;\;\;E_{n}^{2}\left(x\right)=\;\;\mathrm{Tr}\;\left(\Sigma_{n}^{2}\left(x\right)\right)=\;\;\mathrm{Tr}\;\left({𝖫}_{n}^{T}\left(x\right){𝖫}_{n}\left(x\right)\right) \end{array}$$

The matrix ${𝖫}_{n}\;{𝖫}_{n}^{T}$ has the same eigenvalues as the Lyapunov matrix ${𝖫}_{n}^{T}{𝖫}_{n}$ but their eigenvectors are different. The invariants $I_{n}^{\left(k\right)}\left(x\right)$ of the Lyapunov matrix are equal to the sum of the dk products of the eigenvalues and can be computed with the Faddeev–Leverrier algorithm. From a geometrical viewpoint, the invariants are equal to the sum of the squared volumes of the dk parallelotopes whose edges are ${𝖫}_{n}\left(x\right)e_{j}$, and $e_{j}$ are any orthonormal base vectors. According to the Oseledec theorem, by writing the Lyapunov matrix as ${𝖫}_{n}^{T}\left(x\right){𝖫}_{n}\left(x\right)={𝖶}_{n}\left(x\right)\;e^{2n\;\Lambda_{n}\left(x\right)}\;{𝖶}_{n}\left(x\right)$, the diagonal matrix $\Lambda_{n}\left(x\right)$ and the eigenvectors matrix ${𝖶}_{n}\left(x\right)$ have a limit, for n→∞ is equal to Λ(x) and 𝖶(x), respectively. As a consequence, letting $\Lambda \left(x\right)=\;\;\mathrm{diag}\;\left(\lambda_{1}\left(x\right),\ldots ,\lambda_{2d}\left(x\right)\right)$, we have

(4) $$\begin{array}{c} \lim_{n\to \infty }\;\frac{1}{2n}\;\log I_{n}^{\left(k\right)}\left(x\right)=\lambda_{1}\left(x\right)+\lambda_{2}\left(x\right)+\ldots +\lambda_{k}\left(x\right) \end{array}$$

If the map *M* is symplectic, the exponents are pairwise opposite $\lambda_{d+j}=-\lambda_{d-j+1}$. If the map is integrable, then the first *d* eigenvalues of the Lyapunov matrix grow as $n^{2}$, while the last *d* decrease as $n^{-2}$, so that all the Lyapunov exponents vanish. For a generic map, the power law growth or exponential growth with *n* of the first *d* invariants guarantee the classification of the phase space regions of regular and chaotic evolution. Below, we briefly introduce the reversibility error. For a complete description of the indicators and their properties, see Appendix B.

### Reversibility Error

We consider the Backward–Forward process (BF) as one implementation of the Reversibility error indicator. In this case, we iterate *n* times the map with a random perturbation of amplitude ϵ first, and then the inverse map. The recurrence is given by

(5) $$\begin{array}{c} y_{n^{\prime }}=M\left(y_{n^{\prime }-1}\right)+\epsilon \xi_{n^{\prime }}\;1\leq n^{\prime }\leq n\;\;y_{n^{\prime }}=M^{-1}\left(y_{n^{\prime }-1}\right)\;n+1\leq n^{\prime }\leq 2n \end{array}$$

and the linear response is defined by

(6) $$\begin{array}{c} {𝜩}_{2n}^{BF}\left(x\right)=\lim_{\epsilon \to 0}\frac{y_{2n}-x}{\epsilon }=\sum_{k=1}^{n}\;{𝖫}_{k}^{-1}\left(x\right)\;\xi_{k} \end{array}$$

where ${𝖫}_{k}^{-1}$ is the inverse of the matrix ${𝖫}_{k}$ defined by (1). In the absence of perturbation, we are back to the initial condition $y_{2n}\equiv x_{2n}=x$. The covariance matrix of the random vector ${𝜩}_{2n}^{BF}$ is given by

(7) $$\begin{array}{c} \Sigma_{BF\;n}^{2}\left(x\right)=\langle {𝜩}_{2n}^{BF}\left(x\right)\;{\left({𝜩}_{2n}^{BF}\right)}^{T}\rangle =\sum_{n^{\prime }=1}^{n}\;{\left({𝖫}_{n^{\prime }}^{T}\left(x\right){𝖫}_{n^{\prime }}\left(x\right)\right)}^{-1}\; \end{array}$$

The square of the BF reversibility error $E_{BF\;n}$ is defined as the trace of $\Sigma_{BF\;n}^{2}$. The asymptotic limit of the invariants $I_{BF\;\;n}^{\left(k\right)}\left(x\right)$ is the same as (4) for k≤d. The log of the last invariant $I_{BF\;\;n}^{\left(2d\right)}\left(x\right)$ is the Gibbs entropy of the process with the covariance matrix $\Sigma_{BF\;n}^{2}\left(x\right)$ and its asymptotic behavior is the same as the Kolmogorov–Sinai entropy.

If the map is symplectic, then the matrices ${𝖫}_{n^{\prime }}{𝖫}_{n^{\prime }}^{T}$ and ${𝖫}_{n^{\prime }}^{T}{𝖫}_{n^{\prime }}$ are symplectic. Therefore, the trace of ${𝖫}_{n^{\prime }}^{T}{𝖫}_{n^{\prime }}$ and its inverse are equal. In this case, the BF reversibility error is simply related to the Lyapunov error $E_{n}$ as

(8) $$\begin{array}{c} E_{BF\;\;n}^{2}\left(x\right)=\sum_{n^{\prime }=1}^{n}\;E_{n^{\prime }}^{2}\left(x\right) \end{array}$$

Previously, the BF process has been considered with the noise applied to both the B and F iterations. The covariance matrix of the linear response in in this case is given by

(9) $$\begin{array}{c} \Sigma_{BF\;\;n}^{2}\left(x\right)=\frac{1}{2}\langle {𝜩}_{2n}^{BF}\left(x\right)\;\left({𝜩}_{2n}^{BF}\right)\rangle =\frac{1}{2}\;\;I+\sum_{n^{\prime }=1}^{n-1}\;{\left({𝖫}_{n^{\prime }}^{T}\left(x\right){𝖫}_{n^{\prime }}\left(x\right)\right)}^{-1}+\frac{1}{2}\;\;{\left({𝖫}_{n}^{T}\left(x\right){𝖫}_{n}\left(x\right)\right)}^{-1} \end{array}$$

and proof of (9) can be found in [3] Section 2.3. The asymptotic behavior of the invariants of the reversibility error covariance matrix $\Sigma_{nBF}^{2}$ is determined by the positive Lyapunov exponents. Indeed, the limit of ${\left(2t\right)}^{-1}\log\left({𝖨}_{nBF}^{\left(k\right)}\right)$ for t→∞ is the sum of the positive exponents among the first *k*. As a consequence, $\frac{1}{2}\log\left(I_{nBF}^{\left(2d\right)}\right)$, which corresponds to the Gibbs entropy of the BF random process where $I_{nBF}^{\left(2d\right)}=det(\Sigma_{nBF}^{2})$, is the sum of all the positive Lyapunov exponents (the first *d* for a symplectic map), just as the Kolmogorov–Sinai entropy. Notice that the difference with the previous definition and (7) is negligible for a large *n*. Such a definition was initially proposed to compare the reversibility error due to a small random displacement with respect to the reversibility error due to the round off. The Reversibility Error indicator due to round off, denoted by REM, is a numerical implementation of $E_{n}^{R}\left(x\right)$ and numerical equivalence has been proven for simple maps [34].

Denoting with $M_{\epsilon }$ the map evaluated with round off and $M_{\epsilon }^{-1}$ the inverse map evaluated with the round off, the REM indicator is then

(10) $$\begin{array}{c} REM_{BF\;\;n}\left(x\right)=\frac{∥M_{\epsilon }^{\circ \;\;-n}\circ M_{\epsilon }^{\circ \;\;n}\left(x\right)-x∥}{\sqrt{2}\;\;\epsilon } \end{array}$$

The round off is a pseudo-random process in which just one realization is available. When different values for *n* or x are used, it is evident that $REM_{BF\;\;n}$ exhibits significant fluctuations with respect to $E_{BF\;\;n}$, which is the result of an averaging process over the random displacements. No higher invariants can be defined for the reversibility error due to round off.

We can also consider the specular implementation, namely the Forward–Backward $REM_{FB}$, which consists of iterating *n* times first the perturbed inverse map $M^{-1}$ and then the map *M*. For an autonomous symplectic map however, all the invariants are the same as for the BF process. Moreover, for a Hamiltonian flow, equivalence of the FB and BF invariants is a consequence of the time reversal invariance.

The linear approximation given by $y_{n}=x_{n}+\epsilon {𝜩}_{n}$ can be considered; however, since the remainder of order $\epsilon^{2}$ can generically be neglected only for a number of iterations, small with respect to log(1/ϵ), it is of no practical use. The linear response being based on the ϵ→0 limit is valid for any number *n* of iterations.

## 3. Numerical Results for the 2D Billiard

**Figure 1:**
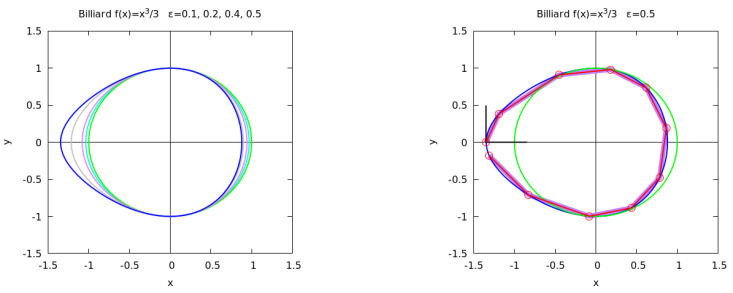
**Left panel**: billiard boundary defined by (A12) with $f\left(x\right)=x^{2}/3$ and ϵ = 0, 0.1, 0.2, 0.4, 0.5 (green, cyan, purple, grey, blue). **Right panel**: rays trajectory for ϵ=0.5 and n=10 (purple line) and the reversed ray trajectory (red line). At the initial point, the tangent and inner normal vectors are shown (black lines).

In this section, we introduce numerical results for a 2D billiard; the reflection map for a convex billiard defined by (A12) with $f\left(x\right)=x^{2}/3$ is presented. The billiard analyzed in this section has a closed boundary for $\epsilon \leq 1/\sqrt{3}∼0.577$ as one can easily show. Indeed, letting $V\left(x\right)=x^{2}/2+\epsilon x^{3}/3$, the maximum occurs for x=−1/ϵ and we require $V\left(-1/\epsilon \right)=1/\left(6\epsilon^{2}\right)\geq 1/2$ to have a closed boundary so that $\epsilon \leq 1/\sqrt{3}$. We have restricted our analysis to ϵ≤/1/2, observing that for ϵ≤0.1, the map is almost integrable; for ϵ=0.2, the area of chaotic regions is significant and already for ϵ=0.4, a large fraction of the phase space is chaotic. We have used the coordinates (ϕ,p) in the phase space even though the map preserves the measure but is not area preserving. We have compared the phase portraits with the Lyapunov error $E_{n}\left(x\right)$, the reversibility error $E_{n}^{BF}$ and the round-off induced reversibility error $REM_{n}^{BF}$, computed according to (A28), (A38) and (A41), respectively. In Figure 1, we show the billiard for:

We have analyzed the orbits for the billiard, comparing them with the color map of the Lyapunov error $E_{n}\left(x\right)$, reversibility error $E_{n}^{BF}\left(x\right)$ and the round-off induced reversibility error $REM_{n}^{BF}\left(x\right)$, where $x={\left(\phi ,p\right)}^{T}$ is chosen on a regular grid of the $N_{g}\times N_{g}$ points. A logarithmic color map is used to show the results. By defining the tangent vector τ(s)=∂r(s)/∂s and the ray velocity as ∥v(s)∥, then the range of the phase ϕ of the position vector is [0,2π], and the range of the momentum p=τ·v is [−1,1]. Since the orbits are symmetric, by changing *p* into −p, the chosen range for *p* is then in the range [0,1]. For a deeper discussion on the properties of the billiard, see Appendix A.

It is important to notice that the indicators give additional information with regard to the phase portrait, since they provide a quantitative measurement of the chaos for an orbit. Additionally, for 4D billiards Poincare’ sections are not available and the indicators are the only methods to investigate the stability of the phase space.

**Figure 2:**
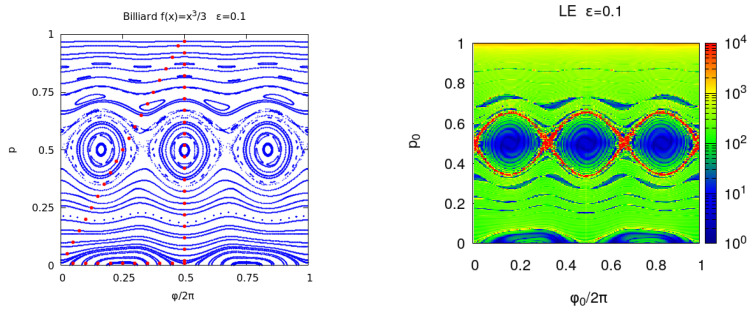
**Left panel**: phase portrait of the billiard with ϵ=0.1. Each orbit is computed for n=1000 and the initial points of each orbit are the red dots. **Right panel**: Lyapunov error $E_{n}\left(x\right)$ where $\phi_{0}/\left(2\pi \right)$ and *p* are chosen in a regular grid of the unit square with $N_{g}\times N_{g}$ points and $N_{g}=200$. The iterations number is n=200 and the results are shown in a logarithmic color scale.

In Figure 2, we compare the phase portrait against the $E_{n}\left(x\right)$ for the billiard with ϵ=0.1. The correspondence between the phase portrait and $E_{n}\left(x\right)$ color plot is good and in both cases, a thin layer of chaotic orbits is seen at the boundary of the main chains of islands. In the interior of the chains of islands, the error tends to be zero because the de-tuning (derivative of the frequency with respect to the action) is low. By approaching the separatrix, the de-tuning grows, diverging on the separatrix itself, where the error growth with *n*, changing from a power law to an exponential one.

**Figure 3:**
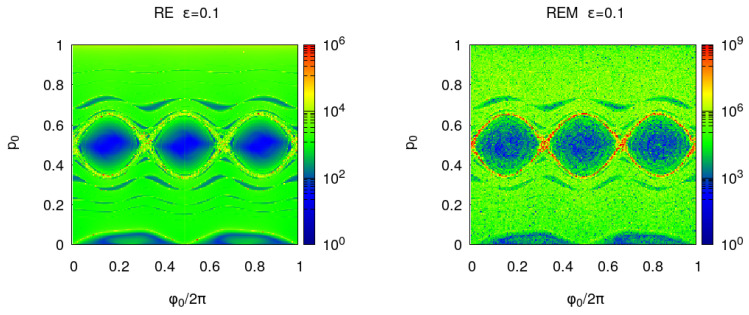
**Left panel**: billiard with ϵ=0.1 reversibility BF error $E_{n}^{BF}\left(x\right)$ where $\phi_{0}/\left(2\pi \right)$ and *p* are chosen in a regular grid of the unit square with $N_{g}\times N_{g}$ points and $N_{g}=200$ and the iterations number is n=200. **Right panel**: round-off induced BF reversibility error $REM_{n}^{BF}\left(x\right)$.

In Figure 3, $E_{n}^{R}\left(x\right)$ and $REM_{n}^{BF}\left(x\right)$ show a similar behavior. REM is similar to the error induced by the small random displacements, but exhibits higher fluctuations because the averaging over the random process is missing. It is worth noticing that ${\left(E_{n}^{R}\right)}^{2}$ constitutes the sum along the orbit of ${\left(E_{n}\right)}^{2}$, provided (s,p) are used as canonical coordinates. The Lyaponov error oscillates with *n* in the regions of quasi-integrable motion and for fixed *n*, oscillations are observed in phase space. These oscillations can be eliminated by using the MEGNO average (see, for instance, Ref. [35]) and they disappear for $E_{n}^{R}\left(x\right)$.

**Figure 4:**
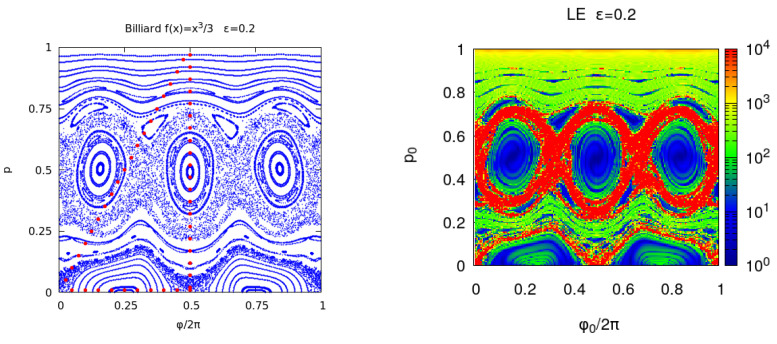
**Left panel**: phase portrait of the billiard with ϵ=0.2. Each orbit is computed for n=1000 and the initial points of each orbit are the red dots. **Right panel**: Lyapunov error $E_{n}\left(x\right)$ where $\phi_{0}/\left(2\pi \right)$ and *p* are chosen in a regular grid of the unit square with $N_{g}\times N_{g}$ points and $N_{g}=200$. The iterations number is n=200 and the results are shown in a logarithmic color scale.

**Figure 5:**
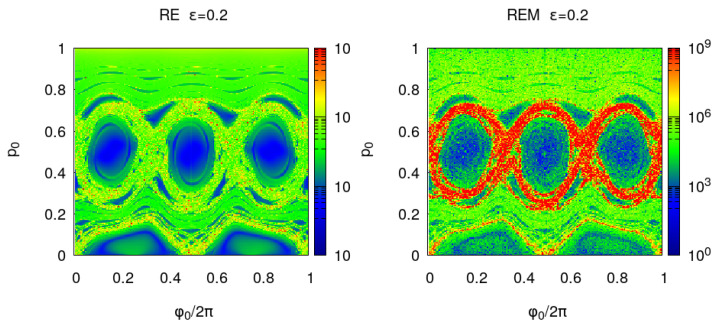
**Left panel**: billiard with ϵ=0.2 reversibility BF error $E_{n}^{BF}\left(x\right)$ where $\phi_{0}/\left(2\pi \right)$ and *p* are chosen in a regular grid of the unit square with $N_{g}\times N_{g}$ points and $N_{g}=200$ and the iterations number is n=200. **Right panel**: round-off induced reversibility error $REM_{n}^{BF}\left(x\right)$.

In Figure 4 and Figure 5, the Poincare section, $E_{n}^{R}\left(x\right)$, $E_{n}\left(x\right)$ and $REM_{n}^{BF}\left(x\right)$ are shown for a larger perturbation ϵ=0.2. The larger deformation with respect to the integrable billiard increases the area of the chaotic orbits. For ϵ=0.3, almost one half of the unit phase space area—in the ($\phi_{0}/2pi$, $p_{0}$) initial coordinates used in the figures—is filled with chaotic orbits and this fraction increases approaching 1, when ϵ tends to the limit value $\epsilon =1/\sqrt{3}$. For a 2D map, the phase space portraits provide the required information on the orbits stability; however, one advantage of the proposed indicators is that they provide a quantitative value to discriminate between regular and chaotic orbits. To explore small details in the phase space, one can analyze a smaller region and increase the number of iterations. Moreover, the indicators become the unique stability analysis tool when the dimensionality of the problem is increased. This is the case for the 3D billiard which leads to a 4D reflection map. In this case, the 2D phase portraits are no longer available.

## 4. Conclusions

We have considered the motion of particles on a 2D billiard with a convex reflecting boundary using a parametrization different from the one proposed by [36]. Our implementation can be easily extended to a 3D billiard. The computation of the arc length *s* on the boundary requires a numerical integration, which can be avoided choosing the phase ϕ of the position vector r rather then the curvilinear abscissa *s*, even though, in this case, the map is only measure preserving. The stability of the orbits has been analyzed using the Lyapunov and Reversibility error fast indicators. Both indicators are invariant with respect to the choice of the initial deviation and of the orthogonal reference frame. We have shown that the logarithm of the second reversibility error invariant is the Gibbs entropy of the random vector defining the deviation from reversibility. The numerical results of the Lyapunov and reversibility error indicators, presented for a selected family of billiards, confirm the reliability of the proposed methods to explore the sensitivity of ray propagation to small random perturbations.

## Figures and Tables

**Figure A1:**
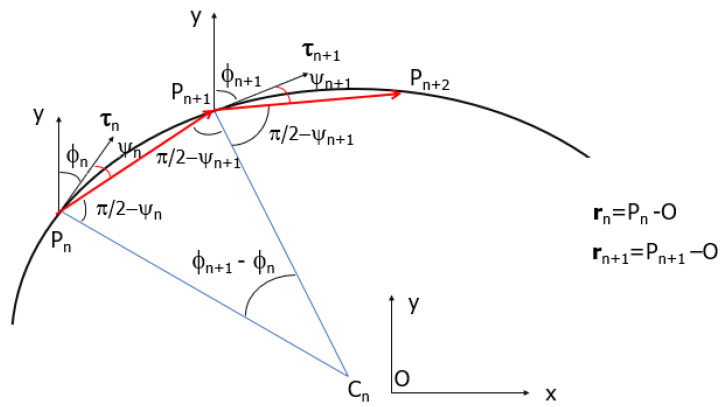
Angles and vectors defining the ray propagation.

## Data Availability

Not applicable.

## Abbreviations

The following abbreviations are used in this manuscript:

2D	Two Dimensional
3D	Three Dimensional
4D	Four Dimensional
EMC	ElectroMagnetic Compatibility
BF	Backward-Forward
REM	Reversibility Error Method
RE	Reversibility Error
LE	Lyapunov Error
MEGNO	Mean Exponential Growth Factor of Nearby Orbits

## Appendix A. Convex Billiards

In this appendix, we consider a plane billiard with a convex boundary and the reflections map, which is area preserving. Letting *s* be the arc length of the boundary, whose length is *ℓ*, the boundary is parameterized by r=r(s), where $r={\left(x,y\right)}^{T}$ and x(s),y(s) are periodic functions of *s* with period *ℓ* [37]. Choose two points $r_{0}=r\left(s_{0}\right)$ and $r_{2}=r\left(s_{2}\right)$ and consider a ray starting at $r_{0}=r\left(s_{0}\right)$, hitting the boundary at r(s) and again at $r_{2}=r\left(s_{2}\right)$. Since $r_{0}$ and $r_{2}$ are given, the intermediate point r is determined by the Maupertuis–Fermat principle which requires the length H(s) of the trajectory to be a minimum

(A1) $$\begin{array}{c} H\left(s\right)=h\left(s_{0},s\right)+h\left(s,s_{2}\right)\;\;h\left(s,s_{0}\right)=∥r\left(s\right)-r\left(s_{0}\right)∥\;\;h\left(s_{2},s\right)=∥r\left(s_{2}\right)-r\left(s\right)∥ \end{array}$$

At any point of the curve *s*, we define the tangent vector τ(s)=∂r(s)/∂s and the inner normal $n\left(s\right)=\tau \left(s\right)\times e_{z}$. Denoting $\dot{x}=dx/ds$ and $\dot{y}=dy/ds$, we have $\tau \left(s\right)=\left(\dot{x},\;\dot{y}\right)$ and $n\left(s\right)=\left(\dot{y},-\dot{x}\right)$. The ray velocity ∥v(s)∥ is constant and is chosen to be equal to 1. Denoting with v(s)≡v(s+0) the velocity of the outgoing ray at the point r(s), we have

(A2) $$\begin{array}{c} v_{0}\equiv v\left(s_{0}\right)=\frac{r\left(s\right)-r_{0}}{h(s,s_{0})}\;\;v\equiv v\left(s\right)=\frac{r_{2}-r\left(s\right)}{h(s_{2},s)} \end{array}$$

Letting $\psi_{0}$ and ψ represent the angles that the ray forms at $s_{0}$ and *s* with the tangents $\tau (s_{0})$ and τ(s), the condition that H(s) is a minimum for $s_{0}$ and $s_{2}$ fixed then gives

(A3) $$\begin{array}{c} \frac{dH(s)}{ds}=\frac{\partial }{\partial s}h\left(s_{0},s\right)+\frac{\partial }{\partial s}h\left(s,s_{2}\right)\\ =\frac{r\left(s\right)-r\left(s_{0}\right)}{h}\cdot \frac{\partial r(s)}{\partial s}-\frac{r\left(s_{2}\right)-r\left(s\right)}{h}\cdot \frac{\partial r(s)}{\partial s}\\ =v_{0}\cdot \tau -v\cdot \tau =\cos\psi_{0}-\cos\psi =0 \end{array}$$

The minimum condition is satisfied for $s=s_{1}$, where $\psi =\psi_{1}$ and setting $v_{1}=v\left(s_{1}\right)$ as the reflection condition $\psi_{1}=\psi_{0}$ is fulfilled. Indeed, the angles that the rays form with the normal are $\theta_{0}=\pi /2-\psi_{0}$ and $\theta_{1}=\pi /2-\psi_{1}$ and the standard condition $\theta_{1}=\theta_{0}$ is recovered. In order to determine the reflection map, we notice that $p_{0}=\cos\psi =v_{0}\cdot \tau_{0}$ and $p_{1}=\cos\psi_{1}=v_{1}\cdot \tau_{1}$ are the momenta conjugated to $s_{0}$ and $s_{1}$. To this end, we consider $h(s_{0},s_{1})$ as the generating function of the map. The moments $p_{0}$ and $p_{1}$ conjugated to $s_{0}$ and $s_{1}$ are given by

(A4) $$\begin{array}{c} p_{1}=\frac{\partial }{\partial s_{1}}h\left(s_{0},s_{1}\right)=v_{1}\cdot \tau_{1}=\cos\psi_{1}\;\;p_{0}=\frac{\partial }{\partial s_{0}}h\left(s_{0},s_{1}\right)=-v_{0}\cdot \tau_{0}=-\cos\psi_{0} \end{array}$$

As a consequence, the map *M* from $x_{0}={\left(s_{0},p_{0}\right)}^{T}$ to $x_{1}={\left(s_{1},p_{1}\right)}^{T}$ generated by $h(s_{0},s_{1})$ is area preserving and s,p are canonical coordinates.

### Appendix A.1. Berry’s Parametrization

A parametrization r=r(ϕ) which allows the analytical computation of the curvilinear abscissa s=s(ϕ) was proposed in [36]. The basic idea is to use ρ=ρ(ϕ) to represent the curve where ρ is the curvature radius and ϕ is the angle which the tangent forms with one of the coordinates’ axes. We choose the origin on the curve at the point where $x=x_{\;\min\;}$ and we move clock-wise. In the case of a circle of radius *R*, we have s=Rϕ and the tangent and the inner normal are given by

(A5) $$\begin{array}{c} r={\left(-\cos\phi ,\sin\phi \right)}^{T}\;\frac{dr}{ds}=\tau ={\left(\sin\phi ,\cos\phi \right)}^{T}\;\frac{d\tau }{ds}=\frac{1}{R}{\left(\cos\phi ,-\sin\phi \right)}^{T}=\frac{r}{R} \end{array}$$

Letting a generic closed curve parameterize according to r=r(ϕ), where ϕ is the angle that the tangent forms with the *y* axis, and ρ(ϕ) the curvature radius, it follows, from the standard formula dτ/ds=n/ρ, where n is the unit inner normal, that

(A6) $$\begin{array}{c} \tau =\frac{dr}{ds}={\left(\sin\phi ,\cos\phi \right)}^{T}\;\;\frac{d\tau }{d\phi }=\frac{d\tau }{ds}\;\frac{ds}{d\phi }=n\frac{1}{\rho }\;\frac{ds}{d\phi } \end{array}$$

Taking the norm of the last equation, where ||dτ/dϕ||=1, we have

(A7) $$\begin{array}{c} \frac{ds}{d\phi }=\rho \left(\phi \right)\;\;r\left(\phi \right)-r\left(0\right)=\int_{0}^{\phi }\tau \left(\phi^{\prime }\right)\frac{ds}{d\phi^{\prime }}\;d\phi^{\prime }=\int_{0}^{\phi }{\left(\sin\phi^{\prime },\;\cos\phi^{\prime }\right)}^{T}\;\rho \left(\phi^{\prime }\right)\;d\phi^{\prime } \end{array}$$

The functions r(ϕ) and ρ(ϕ) are periodic with period 2π and can be expanded in a Fourier series. Since r(0)=r(2π), Equation (A7) implies that the integral of ρcosϕ and ρsinϕ in [0,2π] vanishes. The simplest deformation of the circle is given by

(A8) $$\begin{array}{c} \rho (\phi )=1+\epsilon \cos(2\phi ) \end{array}$$

but any trigonometric polynomial in cos(kϕ) and sin(kϕ) with k≥2 might be considered. In this case, the curve parametrization becomes

(A9) $$\begin{array}{cc} \; & s\left(\phi \right)=\phi +\frac{\epsilon }{2}\;\sin\left(2\phi \right)\\ \; & x\left(\phi \right)=-\cos\phi +\frac{\epsilon }{6}\;\sin\left(3\phi \right)-\frac{\epsilon }{2}\sin\phi \\ \; & y\left(\phi \right)=\sin\phi -\frac{\epsilon }{6}\;\cos\left(3\phi \right)-\frac{\epsilon }{2}\cos\phi \end{array}$$

In order to find the map, we first determine the phase $\phi_{n+1}$ corresponding to the new reflection at $r_{n+1}=r\left(\phi_{n+1}\right)$. To this end, we notice that the direction of the ray emerging at $r_{n}$ is given by the vector $v_{n}$, forming an angle $\psi_{n}$ with $\tau_{n}$ which in turn forms an angle $\phi_{n}$ with the *y* axis. As a consequence, $v_{n}$ forms an angle $\phi_{n}+\psi_{n}$ with the *y* axis. Recalling that $v_{n}=\left(r_{n+1}-r_{n}\right)/∥r_{n+1}-r_{n})∥$, the ratio of the *x* and *y* components is given by

(A10) $$\begin{array}{c} \frac{v_{x\;n}}{v_{y\;n}}=\frac{x_{n+1}-x_{n}}{y_{n+1}-y_{n}}=\frac{\int_{\phi_{n}}^{\phi_{n+1}}\;\rho \left(\phi^{\prime }\right)\;\sin\phi \;d\phi^{\prime }}{\int_{\phi_{n}}^{\phi_{n+1}}\;\rho \left(\phi^{\prime }\right)\;\cos\phi \;d\phi^{\prime }}=\tan\left(\phi_{n}+\psi_{n}\right) \end{array}$$

This equation implicitly determines $\phi_{n+1}$ as a function of $\phi_{n}$ and $\psi_{n}$. Next, we compute $s_{n+1}=s\left(\phi_{n+1}\right)$ and $r_{n+1}=r\left(\phi_{n+1}\right)$ via (A9). The angles entering the reflections at $r_{n}$ and $r_{n+1}$ are shown in Figure A1. The last step is to determine $\psi_{n+1}$; to this end, we consider the triangle whose vertices are $P_{n},\;P_{n+1}$ and $C_{n}$ where the half lines directed along the normals $n_{n}$ and $n_{n+1}$ intersect. Figure A1 shows the displacement vectors with respect to the origin being $r_{n},\;r_{n+1},\;r_{c}$. Taking into account the reflection condition, the angles of this triangle are $\pi /2-\psi_{n}$, $\pi /2-\psi_{n+1}$ and $\phi_{n+1}-\phi_{n}$ from which we obtain

(A11) $$\begin{array}{c} \psi_{n+1}=\phi_{n+1}-\phi_{n}-\;\psi_{n}\;\;p_{n+1}=\cos\psi_{n+1} \end{array}$$

### Appendix A.2. Alternative Parametrization and Reflection Map

We propose another equation for a deformed circle given by

(A12) $$\begin{array}{c} F\left(r\right)=\frac{y^{2}}{2}+\frac{x^{2}}{2}+\epsilon f\left(x\right)-\frac{1}{2}=0\;f\left(x\right)=O\left(x^{3}\right) \end{array}$$

with ϵ>0 and sufficiently small so that the billiard is convex. A parametrization r=r(ϕ) can be easily obtained but requires the solution of an implicit equation. This parametrization was already obtained in [38] and re-derived here. The advantage is that the present form can be easily extended to a deformed sphere. Indeed, at a given point, the curvatures of a surface depend on the two lines traced on it and therefore, on the corresponding tangents which are usually not orthogonal. It is not trivial to represent the curves by assigning the principal curvatures because this implies the knowledge of two families of curves that are mutually orthogonal. The parametrization we propose for the billiard boundary, defined by (A12), is the following:

(A13) $$\begin{array}{c} r=r\left(\phi \right)=r\left(\phi \right){\left(-\cos\phi ,\;\sin\phi \right)}^{T}\;\;\;r\left(\phi \right)=∥r\left(\phi \right)∥ \end{array}$$

In this case, r(ϕ) is the distance from the origin and we denote by ρ(ϕ), the radius of curvature. Setting $\dot{r}=dr/d\phi$, the curvilinear abscissa s(ϕ) is given by

(A14) $$\begin{array}{cc} \; & \frac{dx}{d\phi }=r\sin\phi -\dot{r}\cos\phi \;\frac{dy}{d\phi }=r\cos\phi +\dot{r}\sin\phi \\ \; & ds={\left(r^{2}+{\dot{r}}^{2}\right)}^{1/2}d\phi \;s\left(\phi \right)=\int_{0}^{\phi }\;{\left(r^{2}\left(\phi^{\prime }\right)+{\dot{r}}^{2}\left(\phi^{\prime }\right)\right)}^{1/2}\;d\phi^{\prime } \end{array}$$

The tangent vector ***τ***, the inner normal vector n, and the radius of curvature ρ are given by

(A15) $$\begin{array}{cc} \tau (\phi ) & =\frac{dr}{ds}=\frac{dr}{d\phi }{\left(\frac{ds}{d\phi }\right)}^{-1}=\frac{{\left(r\sin\phi -\dot{r}\cos\phi ,\;r\cos\phi +\dot{r}\sin\phi \right)}^{T}}{{\left(r^{2}\left(\phi \right)+{\dot{r}}^{2}\left(\phi \right)\right)}^{1/2}} \end{array}$$

(A16) $$\rho \left(\phi \right)={\left(\frac{ds}{d\phi }\right)}^{2}{∥\frac{d^{2}r}{d\phi^{2}}-\frac{dr}{d\phi }\frac{d^{2}s}{d\phi^{2}}{\left(\frac{ds}{d\phi }\right)}^{-1}∥}^{-1}\;\;n\left(\phi \right)=\left(\tau_{y}\left(\phi \right),\;\;-\tau_{x}\left(\phi \right)\right)$$

The expressions of ***τ*** and n are still relatively simple, unlike the radius of curvature, which never enters in the computation of the reflection map.

Notice that ϕ is no longer the phase of the vector ***τ*** with respect to the *y* axis. Denoting with $\phi_{\tau }$ the phase of ***τ***, which we choose to be 0 when τ=(1,0) and increasing clock-wise as ϕ, we have

(A17) $$\begin{array}{cc} \tau (\phi ) & ={\left(\sin\phi_{\tau },\;\cos\phi_{\tau }\right)}^{T} \end{array}$$

As a consequence, given ***τ***, its phase is given by

(A18) $$\begin{array}{c} \phi_{\tau }=\arccos\left(\tau_{y}\right)\;\mathrm{if}\;\tau_{x}\geq 0\;\;\phi_{\tau }=2\pi -\arccos\left(\tau_{y}\right)\;\mathrm{if}\;\tau_{x}<0 \end{array}$$

When the equation of the billiard boundary is given by (A13), we determine r=r(ϕ) and $\dot{r}=\dot{r}\left(\phi \right)$ by solving the equations

(A19) $$\begin{array}{c} r={\left(1-2\epsilon f\left(-r\cos\phi \right)\right)}^{1/2}\;\;\;\dot{r}=\frac{\epsilon }{r}\;f^{\prime }\left(-r\cos\phi \right)\left(\dot{r}\cos\phi -r\sin\phi \right) \end{array}$$

iteratively staring with r=1 and $\dot{r}=0$. At first order, $r\left(\phi \right)=1-\epsilon f\left(-\cos\phi \right)+O\left(\epsilon^{2}\right)$. The solution r=r(ϕ) is obtained iteratively and the convergence is very fast for ϵ<<1. The iterations compute the fixed point stops when machine accuracy is reached. Then, s=s(ϕ) is obtained by numerical integration according to (A14).

To compute the reflection map, we start from a point $r_{0}$ with ray velocity $v_{0}$. At the point $r_{0}$, the tangent and inner normal vectors are $\tau_{0}$ and $n_{0}$. We define the phase of a vector b with respect to a vector a as the angle between a and b counted clock-wise and its range is [0,2π]. To the initial position, we associate, according to (A13), the angle $\phi_{0}$ defined as the phase of $-r_{0}$ with respect to the *x* axis. To the initial tangent vector, we associate, according to (A18), the angle $\phi_{\tau \;\;0}$ defined as the phase of $\tau_{0}$ with respect to the *y* axis. If $y_{0}=0$ and $x_{0}<0$, we have $\phi_{0}=\phi_{\tau \;\;0}=0$. The phase of $v_{0}$ with respect to $\tau_{0}$ is $\psi_{0}$ and its range is [0,π] whereas the range of $\phi_{0}$ and $\phi_{\tau \;\;0}$ is [0,2π]. As a consequence, the phase of $v_{0}$ with respect to the *y* axis is $\phi_{\tau \;\;0}+\psi_{0}\;\mathrm{mod}\;2\pi$. The tangential component of the velocity is $p_{0}=v_{0}\cdot \tau_{0}\equiv \cos\psi_{0}$ and if $p_{0}>0$, the reflection is clock-wise; if $p_{0}<0$, it is counter clock-wise. Alternatively, we can start with $\left(\phi_{0},\;p_{0}\right)$ and determine the vectors $r_{0},\;v_{0}$.

After *n* iterations, the point $r_{n}$ on the boundary is reached and the velocity of the outgoing ray is $v_{n}$. The angles $\phi_{n},\;\phi_{\tau \;\;n},\;\psi_{n}$ and the momentum $p_{n}=\cos\psi_{n}$ are determined and eventually the curvilinear abscissa $s_{n}$ is obtained with a numerical integration.

To compute the reflection map, we determine first the unique intersection $r_{n+1}$ of the ray emerging from $r_{n}$ with the boundary. Letting $r\left(t\right)=r_{n}+tv_{n}$, we obtain the unique positive solution of F(r(t))=0 by iteratively solving the equation

(A20) $$\begin{array}{c} t=-2v_{n}\cdot r_{n}-2\epsilon \frac{f\left(x_{n}+t\;v_{x\;n}\right)-f\left(x_{n}\right)}{t} \end{array}$$

As a consequence, we have $r_{n+1}=r_{n}+t_{*}v_{n}$. The phase $\phi_{n+1}$ of $-r_{n+1}$ with respect to the *x* axis, according to (A13), is given by

(A21) $$\begin{array}{cc} \; & \phi_{n+1}=\arccos\left(-\frac{x_{n+1}}{r_{n+1}}\right)\;\mathrm{if}\;y_{n}\geq 0\\ \; & \phi_{n+1}=2\pi -\arccos\left(-\frac{x_{n+1}}{r_{n+1}}\right)\;\mathrm{if}\;y_{n}<0 \end{array}$$

We compute the tangent vector and inner normal vector $\tau_{n+1}=\tau \left(\phi_{n+1}\right)$ and $n_{n+1}=n\left(\phi_{n+1}\right)$ according to (A16). The direction of the emerging ray is computed using the reflection condition:

(A22) $$\begin{array}{cc} v_{n+1} & =v_{n}-2\left(v_{n}\cdot n_{n+1}\right)\;n_{n+1} \end{array}$$

(A23) $$\begin{array}{cc} p_{n+1} & =v_{n+1}\cdot \tau_{n+1}=v_{n}\cdot \tau_{n+1} \end{array}$$

From the momentum $p_{n+1}=v_{n}\cdot \tau_{n+1}$, we determine the phase $\psi_{n+1}=\arccos\left(p_{n+1}\right)$ of $v_{n+1}$ with respect to $\tau_{n+1}$. Recalling that the phase of $v_{n+1}$ with respect to the *y* axis is $\phi_{\tau \;\;n+1}+\psi_{n+1}$, we can check that

(A24) $$\begin{array}{c} v_{n+1}={\left(\sin\left(\phi_{\tau \;\;n+1}+\psi_{n+1}\right),\;\;\cos\left(\phi_{\tau \;\;n+1}+\psi_{n+1}\right)\right)}^{T} \end{array}$$

We recall that the map $\left(\phi_{n+1},p_{n+1}\right)=M\left(\phi_{n},p_{n}\right)$ is measure preserving.

The map $\left(s_{n+1},p_{n+1}\right)=M\left(s_{n},p_{n}\right)$ is symplectic (i.e., area preserving) and can be obtained from the previous map by computing the function s=s(ϕ) according to (A14). This can be done through achieving machine accuracy by storing the results of an integration on a sufficiently fine uniform grid by using Simpson or Gauss integration schemes in each interval, and a linear interpolation.

Finally, the strategy is the following: given an initial value $\phi_{0}$ and $p_{0}$, we determine $r_{0}$ and $v_{0}$. Notice the component of $v_{0}$ along the inner normal is positive ${\left(1-p_{0}^{2}\right)}^{1/2}$ so that $v_{0}$ always points towards an inner point of the billiard. As a consequence, the ray is reflected at a point $r_{1}$ on the boundary, the motion being clock-wise if $p_{0}>0$ and counter clock-wise if $p_{0}<0$. After *N* reflections, the point $r_{N}$ is reached. The reversed trajectory $r{*}_{n}$ is obtained by choosing $r_{0}^{*}=r_{N}$ and $v_{0}^{*}=-v_{N-1}$. It is not difficult to show that $r_{n}^{*}=r_{N-n}$ and $v_{n}^{*}=-v_{N-n-1}$. As a consequence, we have $\phi_{n}^{*}=\phi_{N-n}$ and for the momentum,

(A25) $$\begin{array}{c} p_{n}^{*}=v_{n}^{*}\cdot \tau_{n}^{*}=-v_{N-n-1}\cdot \tau_{N-n}=-v_{N-n}\cdot \tau_{N-n-1}=-p_{N-n} \end{array}$$

These relations imply that $\phi_{N}^{*}=\phi_{0}$ and $p_{N}^{*}=-p_{0}$ and prove that the initial condition is recovered after *N* iterations of the inverse map. If the map is slightly perturbed, then, after *N* iterations of the map, the initial conditions are no longer recovered and the deviation from reversibility is measured by ${\left({\left(\phi_{N}^{*}-\phi_{0}\right)}^{2}+({\left(p_{N}^{*}+p_{0}\right)}^{2}\right)}^{1/2}$.

## Appendix B. Lyapunov and Reversibility Error Indicators

In this appendix, we derive the equivalence between the Lyapunov and reversibility error indicators. Those indicators have been previously defined and used to analyze the stability of other Hamiltonian systems (see, for instance, Ref. [34]). Those indicators measure the growth of a small random perturbation induced on an initial condition or at each reflection. In the latter case, it is convenient to perturb the orbit during the first *n* iterations and integrate the unperturbed inverse map.

These indicators are computed according to the linear response theory, namely when the limit of the perturbation ϵ tends to 0. For this reason, the linear approximation is valid only for a limited number of iterations *n*. The number *n* of iterations up to which the linear approximation is valid depends on x and ϵ and uniform estimates are typically n∼log(1/ϵ). The linear response is valid for any iteration number *N* since it involves the limit to zero of the noise amplitude ϵ. Given a symplectic or measure preserving map M(x), defined in $R^{2d}$, or a compact manifold of dimension 2d, we denote with $x_{n}$ the point of the orbit obtained by iterating the map *n* times, starting from an initial condition x, and by ${𝖫}_{n}\left(x\right)$, its tangent map. Denoting the composition of the map with $M^{\circ 2}\left(x\right)=M\circ M\left(x\right)\equiv M\left(M\left(x\right)\right)$ we have

(A26) $$\begin{array}{cc} \; & x_{n+1}=M\left(x_{n-1}\right)\;\;\;x_{0}=x\;\;x_{n}=M^{\circ n}\left(x\right)\\ \; & {𝖫}_{n+1}\left(x\right)=DM\left(x_{n}\right)\;{𝖫}_{n}\left(x\right)\;{𝖫}_{0}=I\;\;{𝖫}_{n}\left(x\right)=DM^{\circ n}\left(x\right)\\ \; & {\left(DM\right)}_{ij}\left(x\right)=\frac{\partial }{\partial x_{j}}\;M_{i}\left(x\right) \end{array}$$

Consider a nearby initial condition y=x+ϵξ where ***ξ*** is a random vector with a zero mean and unit covariance matrix $\langle \;\xi \;\xi^{T}\;\rangle =I$. The orbit $y_{n}=M\left(y_{n-1}\right)$ is compared with the unperturbed orbit $x_{n}=M\left(x_{n-1}\right)$ and the linear response is given by a random vector ${𝜩}_{n}$ defined by

(A27) $$\begin{array}{cc} \; & {𝜩}_{n}\left(x\right)=\lim_{\epsilon \to 0}\;\frac{y_{n}-x_{n}}{\epsilon }=\lim_{\epsilon \to 0}\;\frac{M^{\circ n}\left(x+\epsilon \xi \right)-M^{\circ n}\left(x\right)}{\epsilon }=DM^{\circ n}\left(x\right)\;\xi \equiv {𝖫}_{n}\left(x\right)\;\xi \end{array}$$

The square of $E_{n}\left(x\right)$ is defined as the trace of the covariance matrix:

(A28) $$\begin{array}{cc} \; & \Sigma_{n}^{2}\left(x\right)=\langle \;{𝜩}_{n}\left(x\right)\;{𝜩}_{n}^{T}\left(x\right)\;\rangle ={𝖫}_{n}\left(x\right)\;{𝖫}_{n}^{T}\left(x\right)\\ \; & E_{n}{\left(x\right)}^{2}=\;\;\mathrm{Tr}\;\left(\Sigma_{n}^{2}\left(x\right)\right)=\;\;\mathrm{Tr}\;\left({𝖫}_{n}^{T}\left(x\right){𝖫}_{n}\left(x\right)\right) \end{array}$$

The matrix ${𝖫}_{n}\;{𝖫}_{n}^{T}$ has the same eigenvalues of the Lyapunov matrix ${𝖫}_{n}^{T}{𝖫}_{n}$ but the eigenvectors are different. The invariants $I_{n}^{\left(k\right)}\left(x\right)$ of ${𝖫}_{n}\;{𝖫}_{n}^{T}$ or ${𝖫}_{n}^{T}\;{𝖫}_{n}$ are the sum of the dk products of the eigenvalues. From a geometric viewpoint, the invariant $I_{n}^{\left(k\right)}$ is the sum of the squared volumes of the dk parallelotopes, whose edges are all the *k* distinct choices among the vectors ${𝖫}_{n}e_{1},\ldots ,{𝖫}_{n}e_{2d}$, having denoted with $e_{j}$ the base vectors ${\left(e_{j}\right)}_{i}=\delta_{ij}$. From the polar decomposition ${𝖫}_{n}={𝖱}_{n}\;e^{n\;\Lambda (n)}\;{𝖶}_{n}^{T}$, where ${𝖱}_{n}$ and ${𝖶}_{n}$ are orthogonal matrices, and the fact that Λ(n) is a real diagonal matrix whose elements are $\lambda_{1}\left(n\right)\geq \lambda_{2}\left(n\right)\geq \ldots \geq \lambda_{2d}\left(n\right)$, is it possible to show that

(A29) $$\begin{array}{c} {𝖫}_{n}^{T}{𝖫}_{n}={𝖶}_{n}\;e^{2n\;\Lambda (n)}\;{𝖶}_{n}^{T}\;\;I_{n}^{\left(k\right)}=\sum_{j_{1}<j_{2}<\ldots <j_{k}}\;\exp\left(\;2n\;\left(\lambda_{j_{1}}\left(n\right)+\ldots +\lambda_{j_{k}}\left(n\right)\;\right)\right) \end{array}$$

If the matrix is symplectic, letting $r^{T}=\left(q,p\right)$ with $q=(q_{1},\ldots ,q_{d})$ and $p=(p_{1},\ldots ,p_{d})$, we have $\lambda_{d+j}\left(n\right)=-\lambda_{d-j+1}\left(n\right)$. The first invariant is $I_{n}^{\left(1\right)}=\;\;\mathrm{Tr}\;\left({𝖫}_{n}^{T}{𝖫}_{n}\right)=E_{n}{\left(x\right)}^{2}$ and the next invariants can be recursively computed with the Faddeev–Leverrier formula, involving the traces of the powers of ${𝖫}_{n}^{T}{𝖫}_{n}$. The last invariant is given by the determinant. Since the eigenvalues $\lambda_{j}\left(n\right)$ have a limit $\lambda_{j}$ for n→∞, the asymptotic limit of the invariants is given by

(A30) $$\begin{array}{c} \lim_{n\to \infty }\;\frac{1}{2n}\;\log I_{n}^{\left(k\right)}\left(x\right)=\lambda_{1}\left(x\right)+\lambda_{2}\left(x\right)+\ldots +\lambda_{k}\left(x\right) \end{array}$$

If a symplectic map *M* is integrable, the eigenvalues grow according to a power law

(A31) $$\begin{array}{c} e^{2n\;\lambda_{j}\left(n\right)}∼\left(1+\alpha_{j}^{2}\;n^{2}\right)\;\;e^{2n\;\lambda_{d+j}\left(n\right)}∼\frac{1}{\left(1+\alpha_{d-j+1}^{2}\;n^{2}\right)}\;\;1\leq j\leq d \end{array}$$

As a consequence, the invariants behave as $I^{\left(k\right)}\left(n\right)∼n^{2k}$ for 1≤k≤d. In the fully chaotic regions, all the first *d* Lyapunov exponents are positive; in the regions of regular motion, they vanish.

### Appendix B.1. Reversibility Error

We consider the Backward–Forward process (BF), in which at each iteration of the reflection map, we add a random perturbation of amplitude ϵ up to the iteration *n* and then iterate *n* times with the inverse map. The linear response ${𝜩}_{n}^{BF}$ for the BF process is obtained by first computing the perturbed orbit:

(A32) $$\begin{array}{cc} \; & y_{n^{\prime }}=M\left(y_{n^{\prime }-1}\right)+\epsilon \xi_{n^{\prime }}\;1\leq n^{\prime }\leq n\\ \; & y_{n^{\prime }}=M^{-1}\left(y_{n^{\prime }-1}\right)\;n+1\leq n^{\prime }\leq 2n \end{array}$$

and observing that the unperturbed orbit, after 2n iterations, comes back to the initial condition $x_{2n}=M^{\circ -n}\circ M^{\circ n}\left(x\right)=x$:

(A33) $$\begin{array}{c} {𝜩}_{2n}^{BF}\left(x\right)=\lim_{\epsilon \to 0}\frac{y_{2n}-x}{\epsilon }=\sum_{k=1}^{n}\;{𝖫}_{k}^{-1}\left(x\right)\;\xi_{k} \end{array}$$

The proof of this relation can be easily obtained by observing that ${𝜩}_{n^{\prime }}$ satisfies the following linear recurrence equation

(A34) $$\begin{array}{cc} \; & {𝜩}_{n^{\prime }}^{BF}\left(x\right)=\lim_{\epsilon \to 0}\frac{M\left(x_{n^{\prime }-1}\right)+\epsilon \xi_{n^{\prime }-1}-M\left(x_{n^{\prime }-1}\right)}{\epsilon }=DM\left(x_{n^{\prime }-1}\right)\;{𝜩}_{n^{\prime }-1}^{BF}\left(x\right)+\xi_{n^{\prime }}\\ \; & {𝜩}_{0}=0\;1\leq n^{\prime }\leq n \end{array}$$

for $n^{\prime }\leq n$ so that for $n^{\prime }=n$ we obtain

(A35) $$\begin{array}{c} {𝜩}_{n}^{BF}\left(x\right)=\xi_{n}+\sum_{k=1}^{n-1}\;DM\left(x_{n-1}\right)DM\left(x_{n-2}\right)\cdots DM\left(x_{k}\right)\;\xi_{k}=\xi_{n}+\sum_{k=1}^{n-1}\;{𝖫}_{n}\left(x\right)\;{𝖫}_{k}^{-1}\left(x\right)\;\xi_{k} \end{array}$$

Since there is no perturbation when iterating with the inverse map $M^{-1}$ for $n+1\leq n^{\prime }\leq 2n$, we have

(A36) $$\begin{array}{c} {𝜩}_{2n}^{BF}\left(x\right)=\lim_{\epsilon \to 0}\frac{M^{-n}\left(y_{n}\right)-M^{-n}\left(x_{n}\right)}{\epsilon }=DM^{-n}\left(x_{n}\right)\;{𝜩}_{n}^{BF}\left(x\right)={𝖫}_{n}^{-1}\left(x\right){𝜩}_{n}^{BF}\left(x\right) \end{array}$$

Indeed, differentiating $M^{-n}\circ M^{n}\left(x\right)=x$, we obtain $DM^{-n}\left(x_{n}\right)\;DM^{n}\left(x\right)=𝖨$ so that $DM^{-n}\left(x_{n}\right)={𝖫}_{n}^{-1}\left(x\right)$. Replacing (A36) into (A34), the equation (A33) follows. The covariance matrices for the BF processes are given by

(A37) $$\begin{array}{c} {\Sigma_{n}^{BF}}^{\;\;2}\left(x\right)=\langle {𝜩}_{2n}^{BF}\left(x\right){𝜩}_{2n}^{BF}\left(x\right)\rangle =\sum_{n^{\prime }=1}^{n}\;{𝖫}_{n^{\prime }}^{-1}\left(x\right)\;{\left({𝖫}_{n^{\prime }}^{-1}\left(x\right)\right)}^{-T}=\sum_{n^{\prime }=1}^{n}\;{\left({𝖫}_{n^{\prime }}^{T}\left(x\right){𝖫}_{n^{\prime }}\left(x\right)\right)}^{-1}\; \end{array}$$

The square of the $E_{n}^{R}\left(x\right)$ is defined as the first invariant, namely, the trace of the covariance matrix $\Sigma_{n}^{BF}$:

(A38) $$\begin{array}{c} {E_{n}^{BF}}^{\;\;2}\left(x\right)=\;\;\mathrm{Tr}\;\left({\Sigma_{n}^{BF}}^{\;\;2}\left(x\right)\right)=\sum_{n^{\prime }=1}^{n}\;\;\;\mathrm{Tr}\;\;\left({\left({𝖫}_{n^{\prime }}^{T}\left(x\right){𝖫}_{n^{\prime }}\left(x\right)\right)}^{-1}\;\right) \end{array}$$

The next invariants of ${\Sigma_{n}^{BF}}^{\;\;2}$ can be computed. If the map is symplectic, then ${𝖫}_{n^{\prime }}{𝖫}_{n^{\prime }}^{T}$ and ${𝖫}_{n^{\prime }}^{T}{𝖫}_{n^{\prime }}$ are symplectic matrices. Therefore, the traces of ${𝖫}_{n^{\prime }}^{T}{𝖫}_{n^{\prime }}$ and its inverse are equal and the RF reversibility error is given by a quadratic average of the $E_{n}\left(x\right)$.

(A39) $$\begin{array}{c} {E_{n}^{BF}}^{\;\;2}\left(x\right)=\sum_{n^{\prime }=1}^{n}\;E_{n}^{2}\left(x\right) \end{array}$$

Previously, the BF process has been considered with the noise applied to both the backward and forward iterations. In this case, the covariance matrix is given by

(A40) $$\begin{array}{c} {\Sigma_{n}^{BF}}^{\;\;2}\left(x\right)=\frac{1}{2}\langle {𝜩}_{2n}^{BF}\left(x\right)\;\left({𝜩}_{2n}^{BF}\right)\rangle =\frac{1}{2}\;\;I+\sum_{n^{\prime }=1}^{n-1}\;{\left({𝖫}_{n^{\prime }}^{T}\left(x\right){𝖫}_{n^{\prime }}\left(x\right)\right)}^{-1}+\frac{1}{2}\;\;{\left({𝖫}_{n}^{T}\left(x\right){𝖫}_{n}\left(x\right)\right)}^{-1} \end{array}$$

A proof of (A40) can be found in [3] Section 2.3. This definition, whose difference with the previous one (A38) is negligible for large *n*, was initially proposed to compare $E_{n}^{R}\left(x\right)$, due to a small random displacement, against REM. Denoting with $M_{\epsilon }$ the map and with $M_{\epsilon }^{-1}$ its inverse, both computed by including the round-off, then the REM is defined by

(A41) $$\begin{array}{c} REM_{n}^{BF}\left(x\right)=\frac{∥M_{\epsilon }^{\circ \;\;-n}\circ M_{\epsilon }^{\circ \;\;n}\left(x\right)-x∥}{\sqrt{2}\;\;\epsilon } \end{array}$$

The round-off is a pseudo-random process of which just one realization is available. It is evident that, as opposed to $E_{n}^{BF}\left(x\right)$, $REM_{n}^{BF}$, it exhibits significant fluctuations when *n* or x vary. No higher invariants can be defined for the reversibility error due to round-off.

The specular process FB, in which we first iterate *n* times the perturbed inverse map $M^{-1}$ and then the map *M*, can be considered. The previous formulae hold where ${𝖫}_{n^{\prime }}\left(x\right)=DM^{n^{\prime }}\left(x\right)$ is replaced with ${𝖫}_{-n^{\prime }}\left(x\right)=DM^{-n^{\prime }}\left(x\right)=DM^{n^{\prime }}\left(M^{-n^{\prime }}\right))={\left({𝖫}_{n^{\prime }}\left(x_{-n^{\prime }}\right)\right)}^{-1}$ and therefore, the covariance matrix for the FB process is

(A42) $$\begin{array}{c} {\Sigma_{n}^{FB}}^{\;\;2}\left(x\right)=\sum_{n^{\prime }=1}^{n}\;{\left({𝖫}_{-n^{\prime }}^{T}\left(x\right){𝖫}_{-n^{\prime }}\left(x\right)\right)}^{-1}\;=\;\sum_{n^{\prime }=1}^{n}\;{𝖫}_{n^{\prime }}\left(x_{-n^{\prime }}\right){𝖫}_{n^{\prime }}^{T}\left(x_{-n^{\prime }}\right)\;x_{-n^{\prime }}=M^{-n^{\prime }}\left(x\right) \end{array}$$

This formula can be proved just as the formula (A40) for the BF covariance matrix. One should take into account exchanging F and B amounts to change the map *M* with $M^{-1}$ so that ${𝖫}_{n^{\prime }}^{T}{𝖫}_{n^{\prime }}$ is replaced by ${𝖫}_{-n^{\prime }}^{T}{𝖫}_{-n^{\prime }}$ since ${𝖫}_{n^{\prime }}=DM^{\circ n^{\prime }}$ and ${𝖫}_{-n^{\prime }}=DM^{\circ \;-n^{\prime }}\equiv D{\left(M^{-1}\right)}^{\circ n^{\prime }}$. For a symplectic map, the BF and FB covariance matrix are equivalent, provided that one exchanges the sign on the momentum, whereas for a dissipative map, they are intrinsically different. In this case, their different behavior reflects the break-up of the time reversal invariance for the unperturbed system. As a consequence, for a symplectic map, the FB reversibility error is related to the Lyapunov error by

(A43) $$\begin{array}{c} {E_{n}^{FB}}^{\;\;2}\left(x\right)=\sum_{n^{\prime }=1}^{n}\;E_{n}^{2}\left(x_{-n^{\prime }}\right) \end{array}$$

The corresponding round-off induced reversibility error REM is defined by iterating *n* times the inverse map $M_{\epsilon }^{-1}$ first, and then the map $M_{\epsilon }$. If x belongs to an ergodic component, then the asymptotic behavior of $E_{n}\left(x\right)$ is the same for any x except for a subset of zero measure. As a consequence, the asymptotic of $E_{n}^{BF}\left(x\right)$ and $E_{n}^{FB}\left(x\right)$ is the same almost everywhere. This is the reason why, for a symplectic map, we can ignore the FB error and consider just the BF reversibility error, which we usually denoted with $E_{n}^{R}\left(x\right)$.
